# Clinical Features of Intra-Abdominal Abscess and Intestinal Free-Wall Perforation in Korean Patients with Crohn’s Disease: Results from the CONNECT Study

**DOI:** 10.3390/jcm10010116

**Published:** 2020-12-31

**Authors:** Seok-Hoo Jeong, Ja Sung Choi, Jin Woo Kim, Hee Man Kim, Hyun-Soo Kim, Jong Pil Im, Joo Sung Kim, You Sun Kim, Jae Hee Cheon, Won Ho Kim, Byong Duk Ye, Young-Ho Kim, Dong Soo Han

**Affiliations:** 1 Department of Internal Medicine, Catholic Kwandong University International St. Mary’s Hospital, Incheon 22711, Korea; ssukoo@naver.com (S.-H.J.); cjs0123@hanmail.net (J.S.C.); 2 Department of Internal Medicine, Yonsei University Wonju College of Medicine, Wonju 26526, Korea; bluebloood@naver.com (J.W.K.); hyskim@yonsei.ac.kr (H.-S.K.); 3 Department of Internal Medicine and Liver Research Institute, Seoul National University College of Medicine, Seoul 03080, Korea; jpim0911@snu.ac.kr (J.P.I.); jooskim@snu.ac.kr (J.S.K.); 4 Department of Internal Medicine, Seoul Paik Hospital, Inje University College of Medicine, Seoul 04551, Korea; yousunk69@korea.com; 5 Department of Internal Medicine and Institute of Gastroenterology, Yonsei University College of Medicine, Seoul 03722, Korea; geniushee@yuhs.ac (J.H.C.); kimwonho@yuhs.ac (W.H.K.); 6 Department of Gastroenterology, University of Ulsan College of Medicine, Asan Medical Center, Seoul 05505, Korea; bdye@amc.seoul.kr; 7 Department of Internal Medicine, Samsung Medical Center, Sungkyunkwan University School of Medicine, Seoul 03181, Korea; younghokim@skku.edu; 8 Department of Internal Medicine, Hanyang University College of Medicine, Guri 11923, Korea; hands@hanyang.ac.kr

**Keywords:** Crohn’s disease, intra-abdominal abscess, perforation

## Abstract

**Background:** In Crohn’s disease (CD), intra-abdominal abscess (IA) and intestinal free-wall perforation (IP) have a common mechanism of transmural inflammation; however, their manifestation is different. **Objective:** This study aimed to investigate differences in the clinical features between IA and IP in Korean patients with Crohn’s disease. **Design**: A retrospective cohort study. **Setting:** Thirty university hospitals and two local hospitals in Korea. **Patients:** Patients who were diagnosed with CD between July 1982 and December 2008 were enrolled. **Main Outcome Measures:** Clinical characteristics of IA and IP. **Results**: Among 1286 patients with CD, 147 (11.4%) had IA and 83 (6.5%) had IP. IA patients were younger than those of IP (24.2 ± 8.6 vs. 30.4 ± 11.1 years, *p* = 0.001). Location and behavior were significantly different between IA and IP (*p* = 0.035 and 0.021). In multivariate analyses, perianal fistula was not associated with increased risk of IA and IP, while intestinal stricture was associated with increased risk of IA (OR: 2.72, *p* < 0.0001) and IP (OR: 2.76, *p* < 0.0001). In subgroup analyses, 55 (36.5%) IA patients were diagnosed at the diagnosis of CD, and 92 (63.5%) during follow-up of CD, while 47 (56.6%) IP patients were diagnosed at the diagnosis of CD, and 36 (43.3%) during follow-up of CD. **Conclusions:** There are several differences in the clinical features of IA and IP in Korean patients with CD. The development mechanism is considered as identical, but further investigation should be needed for clinical implication.

## 1. Introduction

Crohn’s disease (CD) is a chronic disease that can manifest in any part of the gastrointestinal tract and cause significant disability. The small intestine is the most commonly affected part in patients with CD [1,2]. In Asia, including South Korea, the incidence of CD has rapidly increased in the past two decades [3]; however, the incidence of CD in Western countries is still higher than that in Asian countries [4].CD manifests a large variety of symptoms because its location in the gastrointestinal tract is highly variable. The clinical manifestation of CD ranges from mild to severe, even though the symptoms progress gradually [5].

Intra-abdominal abscess (IA) occurs in approximately 10–30% of patients with CD; it often causes serious complications [6,7,8]. There is no standard treatment strategy due to the lack of studies on this specific condition. The current treatment strategy is a combination of antibiotics, percutaneous drainage, and surgery when necessary. The primary risk factors for the failure of medical treatment are abscess size, presence of fistula, and ongoing medications (immunosuppressive agents, steroids) [6,9,10].

Intestinal free-wall perforation (IP) occurs in 1 to 3% of patients with CD. Although this complication is less common during the natural course of the disease, it may manifest as the first complication in 25% of patients [11]. If this is not diagnosed and treated in time, it can easily develop into generalised peritonitis [12]. If this occurs, emergent surgery is often needed to conserve as much of the intestine as possible. In this situation, a selective resection is more effective than large resection to prevent short bowel syndrome and increase the patient’s subsequent quality of life [13]. Frequently, intestinal perforation occurs and immediately covers the surface of a loop, which can lead to IA originating from transmural inflammations. In addition, IP is associated with transmural inflammations [14].

Both these complications have a common development mechanism, but their clinical manifestations are different. To the best of our knowledge, there are no studies on the factors that cause these differences in manifestation. It is important to understand the clinical features of these complications to ensure effective treatment for patients with CD. The aim of the present study was, therefore, to investigate the clinical features of IA and IP among Korean patients with CD and delineate the differences between the two complications.

## 2. Methods

### 2.1. Study Population

The Crohn’s disease clinical network and cohort (CONNECT) study was conducted retrospectively on a nationwide cohort in Korea and was performed by the Korean Association for the Study of Intestinal Diseases [15]. This retrospective multicentre cohort study included patients diagnosed with CD between July 1982 and December 2008 from 30 university hospitals and two local hospitals that cover all major referral centres in Korea. The Ministry for Health, Welfare and Family Affairs and Center for Disease Control in Korea supported this cohort study. CD was diagnosed by gastroenterologists based on clinical, endoscopic, radiological, and histopathologic criteria according to the diagnostic guidelines for CD in Korea [16]. The institutional review board of all 32 hospitals approved the cohort registry and our studies. The requirement for written informed consent was exempted because this study was based on the review of medical records.

### 2.2. Study Design

The demographic and clinical data of patients were retrospectively reviewed using questionnaires and the electronic medical charts provided by each hospital. The location and behaviour of CD were classified according to the Montreal classification [17]. In classifying CD location, L1, L2, L3, and L4 were defined as ileum, colon, ileocolon, and upper gastrointestinal tract location, respectively. In classifying CD behaviour, B1, B2, and B3 were defined as non-stricturing and non-penetrating, stricturing, and penetrating disease, respectively. CD-related complications, including fistulas, abscesses, strictures, and free perforations, were diagnosed based on radiologic or endoscopic diagnostic methods. Stricture was defined as luminal narrowing based on radiologic, endoscopic, or surgical pathologic modalities. Surgery was defined as surgical procedures associated with CD, such as an intestinal resection. IA was defined as a spontaneous abscess formation in the abdominal cavity that was diagnosed using radiologic modalities or confirmed during surgery. IP was defined as a spontaneous perforation of the small or large intestine, which was diagnosed using radiologic or surgical modalities [18]. The control group was defined as patients with CD without abscess and perforation.

Thus, three groups were established for analysis. Among them, the clinical characteristics, including age, sex, onset of CD, location, behaviour, and other complications such as perianal fistula and intestinal stricture, were compared. 

### 2.3. Statistical Analysis

The analyses were conducted using the IBM SPSS version 22 (IBM Co., Armonk, NY, USA) software program. A value of *p* < 0.05 was considered statistically significant. The chi-square test was used to compare the categorical variables. Student’s *t*-test was used to compare the continuous variables among the groups. Multivariate logistic regression analysis was used to investigate the risk factors.

## 3. Results

### 3.1. Clinical Characteristics of Patients with CD

Among a total of 1382 patients with CD, 1286 were included in the present study, while 96 were excluded due to missing data. The patients were categorised according to the complications as control, IA, and IP; the number of patients in each group was 1056 (82.1%), 147 (11.4%), and 83 (6.5%), respectively. 

The clinical characteristics of each group are summarised in Table 1. The mean age was 24.2 ± 8.6 years in the IA group and 27.3 ± 12.4 years in the control group (*p* = 0.009). The presence of perianal fistula at diagnosis and its development after diagnosis was significantly higher in the IA group than in the control group (48.3% vs. 36.2%, *p* = 0.041). The IA group showed a significantly higher prevalence of intestinal stricture at diagnosis and its development after diagnosis than did the control group (37.4% vs. 24.2%, *p* < 0.001). Regarding the behaviour, B3 (penetrating disease) was significantly higher in the IA group than in the control group (13.6% vs. 8.4%, *p* = 0.002).

The mean age was 30.4 ± 11.1 years in the IP group and 27.3 ± 12.4 years in the control group (*p* = 0.073). The IP group showed a significantly higher prevalence of intestinal stricture at diagnosis and its development after diagnosis than the control group (36.1% vs. 24.2%, *p* < 0.001). Regarding the behaviour, B2 (structuring disease) and B3 (penetrating disease) were present in a significantly higher frequency in IP group than in the control group (B2 (8.4% vs. 6.6%), B3 (28.9% vs. 8.4%), *p* < 0.001).

The IP group was younger than the IA group (24.2 ± 8.6 years and 30.4 ± 11.1 years, respectively; *p* = 0.001). There was no difference in the sex ratio between both groups. The presence of perianal fistula at diagnosis and development after diagnosis in the IA group was significantly more frequent than that in the IP group (48.3% vs. 27.7%, *p* = 0.022). There was no difference in the presence of intestinal stricture between both groups. Regarding the behaviour, B2 (structuring disease) and B3 (penetrating disease) were present in a significantly higher proportion in the IP group than in the IA group (B2 (8.4% vs. 5.4%), B3 (28.9% vs. 13.6%), *p* = 0.021). There was a significant difference in the locations between both groups (*p* = 0.035).

### 3.2. Association with Intestinal Stricture and Perianal Fistula

In multivariate analyses with adjustment for age and sex, the development of intestinal stricture after diagnosis was associated with an increased risk of IA (odds ratio (OR) = 2.72, 95% confidence interval (CI) = 1.80–4.10) in Table 2. The development of intestinal stricture after diagnosis was associated with an increased risk of IP (OR = 2.76, 95% CI = 1.57–4.84) in Table 3. Perianal fistula was not associated with IA and IP.

### 3.3. Clinical Characteristics of Patients with Intra-Abdominal Abscess and Free-Wall Perforation 

The mean age of the patients with IA at the diagnosis of CD was significantly higher than that of the patients with IA at the follow-up of CD (*p* = 0.006) in Table 4. There were no differences in the sex, location of lesion, behaviour of lesion, use of steroids, and smoking between the patients with IA at the diagnosis and at the follow-up of CD.

The mean age (*p* = 0.004) and behaviour (*p* = 0.002) were significantly different between patients with IP at the diagnosis and at the follow-up of CD in Table 5. There were no differences in the sex, location of lesion, use of steroids, and smoking between patients with IP at the diagnosis and at the follow-up of CD. 

## 4. Discussion

If deep transmural ulcers are present in patients with CD, it usually leads to complications such as fistula, abscess, and perforation. Therefore, patients with CD frequently require surgery. The incidence of IA was found to be 10–30% in Western countries [19,20,21]. In comparison, the cumulative incidence of abscesses is 9–25% in Japan [22]. A previous study reported that the incidence of IP is relatively higher in Japan than in Western countries (6.5% vs. 1–3%) [18].

Our study identified that the presence of intestinal stricture at development after diagnosis increased the risk of IA and IP (OR = 2.72 and 2.76, 95% CI = 1.80–4.10 and 1.57–4.84). These results are consistent with the results of previous studies that showed that anal lesions and bowel strictures were risk factors for IA or IP [18,23].

Although the exact mechanisms of IA and IP are not known, there are some hypotheses. A common mechanism of IA suggests that inflammatory processes directly involve all the intestinal layers, which then result in ulcer formation. The processes that lead to ulcer formation can also lead to abscesses in the closed space between the intestinal lumen and adjacent organs. Direct seeding may also, rarely, lead to abscess formation: in this process, the circulation of blood via intestinal lesions seeds directly to the distant organs. The most plausible mechanism of IP is that the increasing pressure at the proximal stenotic lumen of the intestine induces bowel distension, which results in perforation of the thin layer with deep ulceration [24,25,26,27]. In addition, bowel ischemia that results in vessel inflammation may induce perforation in patients without dilatations or strictures [28]. We thought that the speed of lesion development, the depth of the ulcer, and the severity of the stenosis would be other factors that determine incidence of IA or IP.

Although the mechanisms of IA and IP might be similar, our study identified some differences in the clinical features of patients with IA and IP. The mean age, behaviour (B2 and B3), and lesion location (L1, L2, and L3) were higher in the IA group than in the IP group. In this regard, we thought that the IA might be a risk factor for IP. A previous study identified that concomitant upper gastrointestinal tract involvement was an associated factor for IA [29]. In addition, concomitant upper gastrointestinal tract involvement was associated with the time of first surgery in CD patients in a United States study population [30]. Therefore, these suggested locations might be associated with the progression of CD and clinical outcomes. 

The mean age in patients with IA and IP at the diagnosis of CD was significantly higher than that in patients with IA and IP at the follow-up of CD. These results may cause a delay in continuing treatment or diagnosis, which then result in severe complications such as IA and IP [31,32,33]. A recent study showed that a delay in diagnosis might have a negative impact on the progression of disease [34]. However, longitudinal studies have shown that a diagnostic delay in CD might influence disease outcomes [7,35]. Therefore, the early diagnosis of CD may be important to monitor its progression, and the clinical outcomes will differ because of early treatment. 

To the best of our knowledge, our study is the first report on the clinical characteristics of IA and IP in patients with CD using data from a large-scale multicentre cohort study in East Asia. However, our study had some limitations due to its retrospective design. First, some of the medical records did not contain complete descriptions of the IA and IP. We reported that 245 (21.1%) of 1161 patients with CD received surgery approached through the abdomen in the other paper [36]. However, a detailed description of surgical methods was not surveyed in our cohort, so that we did not analyzed surgical methods and surgical complications for IA and IP. Second, confounding factors such as smoking and disease activity were not considered due to the lack of relevant information in the medical records. Third, there was a bias regarding overestimation of CD complications due to this being a hospital-based study. Finally, medications including biologic agents were not included for analysis, because some patients were diagnosed with Crohn’s disease simultaneously with IA or IP, and their IA or IP was not associated with medications for CD. 

In conclusion, there are several differences in the clinical features of IA and IP in Korean patients with CD, although these two complications have a common development mechanism. This study identifies that the presence of intestinal stricture at development after diagnosis increases the risk of IA and IP both.

## Figures and Tables

**Table 1:** Clinical characteristics of patients with intra-abdominal abscess (IA) and intestinal free-wall perforation (IP) (*n* = 1286).

	Control	IA	IP	*p* Value		
				Control vs. IA	Control vs. IP	IA vs. IP
*n*	1056 (82.1%)	147 (11.4%)	83 (6.5%)			
Age at diagnosis of CD (years)	27.3 ± 12.4	24.2 ± 8.6	30.4 ± 11.1	0.009	0.073	0.001
Sex (M:F)	739 (70.0%):317 (30.0%)	105 (71.4%):42 (28.6%)	63 (75.9%):20 (24.1%)	0.719	0.255	0.463
Diagnosis of complications				-	-	0.005
At diagnosis of CD	-	55 (37.4%)	47 (56.6%)			
During follow-up of CD	-	92 (62.6%)	36 (43.4%)			
Perianal fistula				0.041	0.399	0.022
No	657 (62.2%)	75 (51.0%)	59 (71.1%)			
Presence at diagnosis	275 (26.0%)	51 (34.7%)	18 (21.7%)			
Development after diagnosis	108 (10.2%)	20 (13.6%)	5 (6.0%)			
unknown	16 (1.5%)	1 (0.7%)	1 (1.2%)			
Intestinal stricture				<0.0001	<0.0001	0.228
No	791 (74.9%)	90 (61.2%)	49 (59.0%)			
Presence at diagnosis	123 (11.6%)	11 (7.5%)	10 (12.0%)			
Development after diagnosis	133 (12.6%)	44 (29.9%)	20 (24.1%)			
Unknown	9 (0.9%)	2 (1.4%)	4 (4.8%)			
Location				0.229	0.065	0.035
L1	156 (14.8%)	15 (10.2%)	20 (24.1%)			
L2	138 (21.5%)	17 (11.6%)	4 (4.8%)			
L3	333 (31.5%)	40 (27.2%)	22 (26.5%)			
L4	13 (1.2%)	2 (1.4%)	2 (2.4%)			
L3 + L4	3 (0.3%)	0 (0%)	0 (0%)			
Unknown	413 (39.1%)	73 (49.7%)	35 (42.2%)			
Behavior				0.002	<0.0001	0.021
B1	423 (40.1%)	37 (25.2%)	15 (18.1%)			
B2	70 (6.6%)	8 (5.4%)	7 (8.4%)			
B3	89 (8.4%)	20 (13.6%)	24 (28.9%)			
Unknown	474 (44.9%)	82 (55.8%)	37 (44.6%)			

**Table 2:** Multivariable analysis for the risk factors of intra-abdominal abscess.

	Intra-Abdominal Abscess		*p* Value
	Odds Ratio	95% CI	
Perianal fistula			
No	1		
Presence at diagnosis	1.41	0.95–2.11	0.088
Development after diagnosis	1.39	0.80–2.42	0.240
Intestinal stricture			
No	1		
Presence at diagnosis	0.85	0.44–1.64	0.618
Development after diagnosis	2.72	1.80–4.10	<0.0001

Excluded unknown (control = 1030, intra-abdominal abscess = 144; adjustment for age and sex).

**Table 3:** Multivariable analysis for the risk factors of intestinal free-wall perforation.

	Intestinal Free-Wall Perforation		*p* Value
	Odds Ratio	95% CI	
Perianal fistula			
No	1		
Presence at diagnosis	0.70	0.38–1.26	0.228
Development after diagnosis	0.57	0.22–1.49	0.254
Intestinal stricture			
No	1		
Presence at diagnosis	1.26	0.62–2.57	0.523
Development after diagnosis	2.76	1.57–4.84	<0.0001

Excluded unknown (*n* = 1030, *n* = 78) adjustment for age and sex.

**Table 4:** Clinical characteristics of patients with intra-abdominal abscess (*n* = 147).

	Intra-Abdominal Abscess		*p* Value
	At Diagnosis of CD	Follow-Up of CD	
*n*	55 (36.5%)	92 (63.5%)	
Age at diagnosis of CD (years)	26.7 ± 9.4	22.7 ± 7.7	0.006
Sex (M:F)	42 (76.4%):13 (26.3%)	63 (68.5%):29 (31.5%)	0.306
Location			0.234
L1	7 (12.7%)	8 (8.7%)	
L2	3 (5.5%)	14 (15.2%)	
L3	18 (32.7%)	22 (23.9%)	
L4	0 (0%)	2 (2.2%)	
Unknown	27 (49.1%)	46 (50.0%)	
Behavior			0.682
B1	12 (21.8%)	25 (27.2%)	
B2	2 (3.6%)	6 (6.5%)	
B3	9 (16.4%)	11 (12.0%)	
Unknown	32 (58.2%)	50 (54.3%)	
Steroid at diagnosis of CD			0.133
Use	8 (14.5%)	22 (23.9%)	
No use	21 (38.2%)	22 (23.9%)	
unknown	26 (47.3%)	48 (52.2%)	
Smoking			0.869
Never	23 (41.8%)	34 (38.8%)	
Current or Ex-smoker	9 (16.4%)	18 (17.6%)	
unknown	23 (41.8%)	40 (43.5%)	

**Table 5:** Clinical characteristics of patients with free-wall perforation (*n* = 83).

	Intestinal Free-Wall Perforation		*p* Value
	At Diagnosis of CD	Follow-Up of CD	
*n*	47 (56.6%)	36 (43.3%)	
Age at diagnosis of CD (years)	33.4 ± 10.9	26.4 ± 10.2	0.004
Sex (M:F)	36 (76.6%):11 (23.4%)	27 (75.0%):9 (25.0%)	0.866
Location			0.030
L1	15 (31.9%)	5 (13.9%)	
L2	3 (6.4%)	1 (2.8%)	
L3	14 (29.8%)	8 (22.2%)	
L4	2 (4.3%)	0 (0%)	
Unknown	13 (27.7%)	22 (61.1%)	
Behavior			0.002
B1	6 (12.8%)	9 (25.0%)	
B2	6 (12.8%)	1 (2.8%)	
B3	20 (42.6%)	4 (11.1%)	
Unknown	15	22 (61.1%)	
Steroid at diagnosis of CD			0.005
Use	7 (14.9%)	7 (19.4%)	
No use	24 (51.1%)	6 (16.7%)	
unknown	16 (34.0%)	23 (63.9%)	
Smoking			0.549
Never	22 (46.8%)	11 (30.6%)	
Current or Ex-smoker	9 (19.2%)	8 (22.2%)	
unknown	16 (34.0%)	17 (47.2%)	

## Data Availability

Data sharing is not applicable to this article.
